# Highly Sensitive Flow Sensor Based on Flexible Dual-Layer Heating Structures

**DOI:** 10.3390/s20226657

**Published:** 2020-11-20

**Authors:** Yu-Chao Yan, Cheng-Yu Jiang, Run-Bo Chen, Bing-He Ma, Jin-Jun Deng, Shao-Jun Zheng, Jian Luo

**Affiliations:** Key Lab of Micro/Nano Systems for Aerospace, Ministry of Education, Northwestern Polytechnical University, Xi’an 710072, China; yanyuchao@mail.nwpu.edu.cn (Y.-C.Y.); jiangcy@nwpu.edu.cn (C.-Y.J.); 2311877145@mail.nwpu.edu.cn (R.-B.C.); dengjj@nwpu.edu.cn (J.-J.D.); zhengshaojun@mail.nwpu.edu.cn (S.-J.Z.); jian.luo@nwpu.edu.cn (J.L.)

**Keywords:** dual-layer structure, hot film, heat conduction insulation, flow shear stress

## Abstract

Hot film sensors detect the flow shear stress based on the forced convection heat transfer to the fluid. Current hot film sensors have been significantly hindered by the relatively low sensitivity due to the massive heat conduction to the substrate. This paper describes the design, fabrication, simulation, and testing of a novel flow sensor with dual-layer hot film structures. More specifically, the heat conduction was insulated from the sensing heater to the substrate by controlling both sensing and guarding heaters working at the same temperature, resulting in a higher sensitivity. The experiment and simulation results showed that the sensitivity of the dual-layer hot film sensor was significantly improved in comparison to the single-layer sensor. Additionally, the dual-layer sensor was designed and fabricated in an integrated, flexible, and miniaturized manner. Its small size makes it an excellent candidate for flow detection.

## 1. Introduction

With the development of micro-machining and flexible electronic technology, flexible micro-sensors have attracted wide attention in the applications of wearable devices [1,2,3,4] and artificial skin [5,6,7]. Previously, flexible sensors mainly focused on the detection of pressure [8,9,10,11] and temperature [12,13,14]. Increasing attention has been paid to flow detection in recent years [15,16,17,18].

Flow shear stress is an important parameter of fluid mechanics, which is directly related to the flow friction on the surface of vehicles [19,20,21]. Based on the principle of forced convection heat transfer, the hot film sensors are widely applied to the detection of flow shear stress. Previously, hot film sensors were generally in a single-layer structure where a large amount of heat was dissipated from the sensing heater to the substrate through heat conduction, weakening the sensors’ sensitivity to the flow shear stress. In order to insulate the heat conduction from the sensing heater to the substrate and improve the sensitivity to the flow shear stress, research on the heat insulation has been widely explored. Ou Y. et al. fabricated a hot film sensor featuring a vacuum cavity under the sensing heater, because the vacuum chamber provided a substrate with low thermal conductivity [22]. Although this type of sensor has been successfully applied to flow measurement, it has been critically hampered by the sensitivity to variance in air pressure. As a new type of heat-insulating scheme, a heat-insulating method with a guarding heater structure appeared. Rustom B. Bhiladvala et al. simulated heat transfer based on a hot film sensor with dual-layer structures, showing that the dual-layer structure was beneficial to improving the sensitivity and response frequency of the hot film sensor [23,24]. However, they did not fabricate the dual-layer hot film sensor or verify the simulation results practically. Gao Nan et al. bonded two overlapped nickel films on the double surfaces of a polyimide (PI) film to obtain a sandwiched hot film sensor and carried out calibrations in a small wind tunnel [25,26]. However, the heat-insulating layer of their sandwiched structure sensor was thick, leading to a large size of the sensor (22 mm in width and 1.75 mm in length) and a compromised heat-insulating effect of the guarding heater. Nevertheless, the approach to develop an integrated and miniaturized dual-layer flexible hot film sensor has rarely been discussed or demonstrated.

In this paper, a micro flexible hot film sensor with dual-layer structure was designed and fabricated. The polyamic acid (PAA) electrical-insulating layer was only 7 μm, and the total thickness of the dual-layer sensor was within 80 μm, which kept the sensor flexible, miniaturized, and able to meet the requirement of flow measurement. Simulations and experiments consolidated the advantages in enhancing sensitivity and reducing heat conduction.

## 2. Fabrication

**Figure 1:**
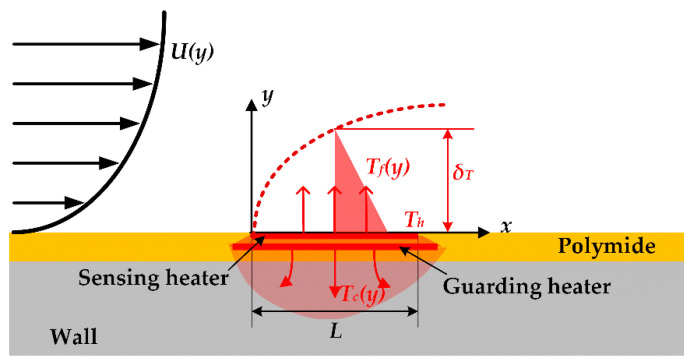
The working principle of the dual-layer hot film sensor.

The hot film sensor detected flow by measuring the velocity gradient in the force convection heat transfer process as shown in Figure 1. The velocity gradient was proportional to the flow shear stress, which could be expressed as

(1) $$\tau =\mu \frac{dU\left(y\right)}{dy},$$

where τ was the flow shear stress, μ was viscosity, and dU(y)/dy was the velocity gradient. The joule heat generated by the sensing heater *Q_tot_* equal to the heat dissipation, and the main methods of heat dissipation are heat conduction to substrate *Q_c_* and heat transfer to fluid *Q_f_*. When the flexible thermal film sensor stays in heat balance, the heat dissipation relationship can be expressed as

(2) $$Q_{tot}=Q_{c}+Q_{f},$$

The heat conduction *Q_c_* of the hot film sensor to the substrate could be expressed as

(3) $$Q_{c}=\lambda_{s}A\frac{\partial T_{c}\left(y\right)}{\partial y},$$

where $\lambda_{s}$ is thermal conductivity, A is the heat exchange area, and $\partial T_{c}\left(y\right)/\partial y$ is the temperature gradient. The sensing heater and guarding heater were operated at the same working temperature, where $\partial T_{c}\left(y\right)/\partial y$ was approximately zero. Therefore, the dual-layer hot film sensor can isolate the heat conduction from the sensing heater to the substrate. For the sensing heater of the dual-layer hot film sensor, the heat dissipation is equal to the heat convection transferring to the fluid.

**Figure 2:**
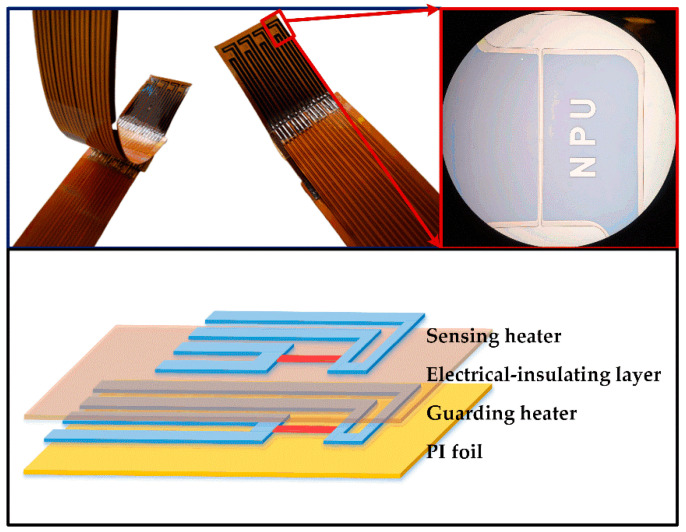
The photograph and structure of the flexible dual-layer hot film sensors.

**Figure 3:**
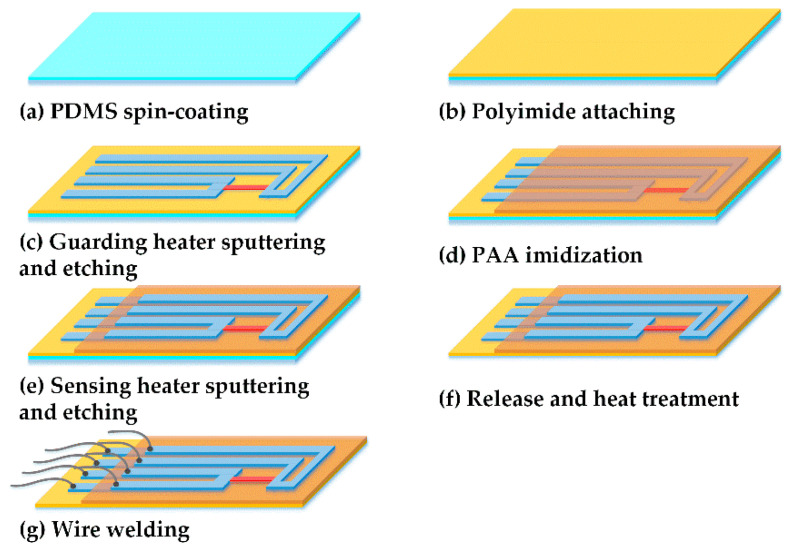
The fabrication processes for a flexible dual-layer hot film sensor.

The dual-layer hot film sensor consisted of a flexible PI foil, a lower guarding heater, a PAA electrical-insulating layer, and an upper sensing heater. The photograph and structure of the flexible dual-layer hot film sensors are shown in Figure 2, and the fabrication processes are presented in Figure 3. The center alignment error of the two-layer structures was less than 0.8 μm, and the total thickness of the dual-layer sensor was within 80 μm.

The guarding heater of the dual-layer sensor was fabricated in the same way as the single-layer hot film sensor [27]. Polydimethylsiloxane (PDMS) was spin-coated as an adhesive between the PI foil and the glass carrier. A nickel film was sputtered onto the PI foil and patterned by photolithography to obtain the guarding heater. PAA was spin-coated between the guarding heater and the sensing heater with a thickness of 7 μm, forming an electrical-insulating layer after the imidization reaction. Then, the sensing heater was fabricated by sputtering secondary nickel film onto the imido PAA and patterned by photolithography again. Next, the flexible dual-layer hot film sensor was peeled off from the glass. A heat treatment was carried out to enhance the adhesion between the guarding heater and the PI foil, which also improved the temperature coefficients of resistance (TCR) of the two-layer heaters. Finally, flexible circuit wires were welded into the two-layer heaters.

The resistance and TCR were two basic parameters of the flexible dual-layer hot film sensors, which were measured in a temperature-test system to ensure that the resistance of the sensor was linear with the temperature as

(4) $$R=R_{20}\left[1+\alpha_{20}\left(T-20\right)\right],$$

where *R* was the resistance of the heater at temperature *T*. *R*_20_ and *α*_20_ were the resistance and TCR at 20 ℃, respectively.

The measurements of basic parameters were carried out at four equidistant temperatures from 20 to 80 °C. Table 1 shows the test results of the heaters’ parameters. “S” represents the sensing heater and “G” represents the guarding heater. A group of “S” and “G” constitutes a dual-layer structured sensor. The correlation coefficients *R*^2^ of the linear fitting were all better than 0.9999, indicating that the resistances of the heaters exhibited excellent linear relationships with the temperatures.

## 3. Experiment

**Figure 4:**
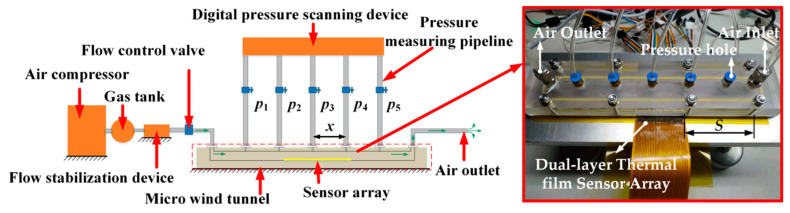
Schematic diagram and photograph of micro-wind tunnel system. The wall static pressures were measured by the pressure scanner. Δ*x* = 4 cm, *h* = 0.535 mm.

The flexible dual-layer hot film sensors were tested in a micro-wind tunnel system as shown in Figure 4. Due to the limited height of the tunnel (0.535 mm), a fully developed flow could be generated in the micro wind tunnel. The flow shear stress was linear to the wall static pressure gradient along the streamwise. The wall static pressures were measured by a pressure scanner. The relationship between the flow shear stress and the wall static pressure along the streamwise can be expressed as

(5) $$\tau =-\frac{h}{2}\frac{dp}{dx}=\frac{h}{2}\frac{p_{5}-p_{1}}{4x},$$

where *p_1_* and *p_5_* are the wall static pressures at the first pressure tap and the fifth pressure tap, *h* is the height of the wind tunnel, and *x* is distance between two adjacent pressure taps. The flexible dual-layer hot film sensors were flush-mounted on the wall of the wind tunnel.

The sensing heater and guarding heater were required to work at the same temperature during the calibrations. Hence, the working temperatures were controlled precisely. However, the common constant temperature drive systems had only two wires, and the wire resistances would affect the control accuracy of the sensor’s working temperature. The sensing heater and guarding heater of the proposed dual-layer hot film sensor were designed with four wires to eliminate the influence of wire resistances on the output voltage. The sensing heater and guarding heater were driven by an Agilent B2962A double-channel constant current source with controllable electric currents, and the electric currents were measured by two Keysight 34465A digital multimeters in real-time. The output voltages of the two-layer heaters were measured by a Dewesoft multichannel data acquisition system. The working resistances and working temperatures of the two-layer heaters could be calculated with the measured output voltages and electric currents. The two-layer heaters worked at the same target temperature of 45 °C by adjusting the drive currents. Calibrations were conducted on single-layer sensors of S1, S2, S3, S4 and dual-layer sensors S1G1, S2G2, S3G3, S4G4, respectively. Specifically, the dual-layer hot film sensors could be regarded as single-layer hot film sensors when the guarding heater did not heat, and the single-layer hot film sensors also worked at 45 °C.

**Figure 5:**
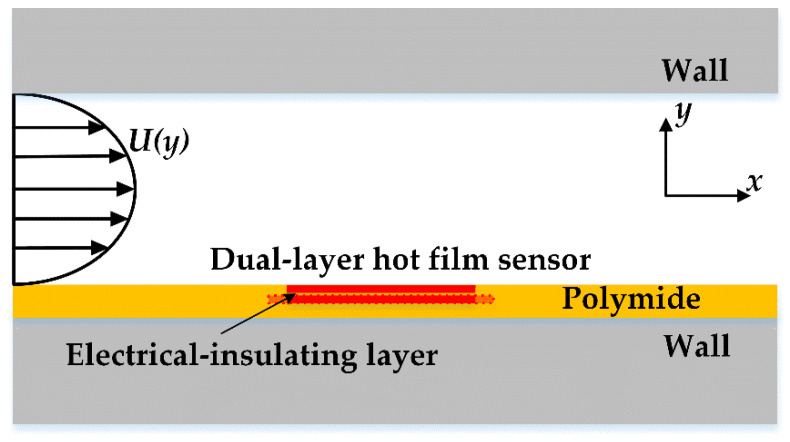
The schematic of simulation model.

Hot film sensors with dual-layer and single-layer structures were simulated by COMSOL software to analyze the heat-insulating effect of the dual-layer structure on the heat conduction from the sensing heater to the substrate. The simulation structural model is shown in Figure 5. The flexible dual-layer hot film sensor was flush-mounted on the wall of the wind tunnel. The length of the sensor along the flow direction was 50 μm. The height of the tunnel was 0.535 mm, therefore a fully developed laminar flow, also known as Poiseuille flow, could be generated through it. The mesh size was determined by the temperature gradient. The average temperature gradient and shear rate were calculated to obtain the heat power and flow shear stress.

## 4. Results and Discussion

**Figure 6:**
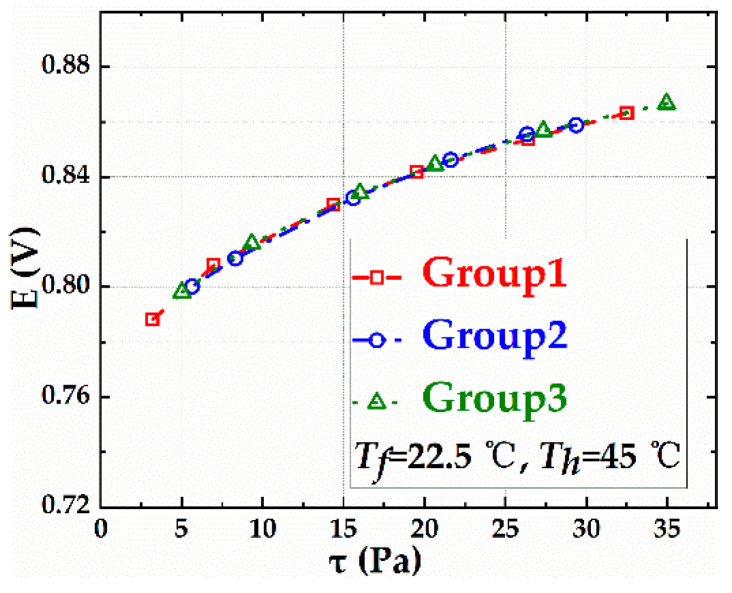
The repeated data of the experimental system with the sensor S1.

Repetitive experiments were conducted three times to evaluate the repeatability of the calibration system with sensor S1. The guarding heater G1 did not heat when calibrating sensor S1, hence, sensor S1 could be regarded as a flexible single-layer hot film sensor. The experimental data of sensor S1 are shown in Figure 6. The repeated data almost overlapped with a repetitive error of 0.13%, demonstrating that the calibration system has high precision.

**Figure 7:**
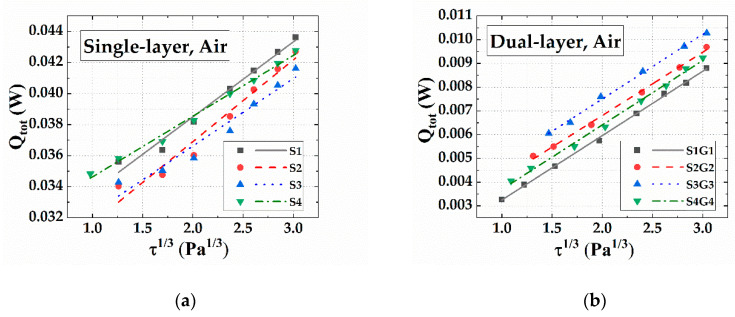
The calibration results of the (**a**) single-layer and (**b**) dual-layer flexible hot film sensors.

The calibration data of single-layer and dual-layer hot film sensors are shown in Figure 7. The total heating powers *Q_tot_* of the sensors are linear to the third power of the flow shear stresses. The total heating powers *Q_tot_* of the dual-layer hot film sensor are far lower than those of the single-layer hot film sensor, demonstrating that the dual-layer structure effectively reduced the heat conduction from the sensing heater to the substrate.

Table 2 shows the average sensitivities of the single-layer and dual-layer hot film sensors. The average sensitivity was the average voltage variation of the sensor with the flow shear stress varying from 0 to 30 Pa. Compared with the single-layer sensors, the dual-layer sensors significantly improved the average sensitivity. For example, the dual-layer sensor S1G1 exhibited an average sensitivity of 9.85 mV/Pa with the flow shear stress changing from 0 to 30 Pa, which improved by 110.47% in comparison with the single-layer sensor S1 (4.68 mV/Pa).

**Figure 8:**
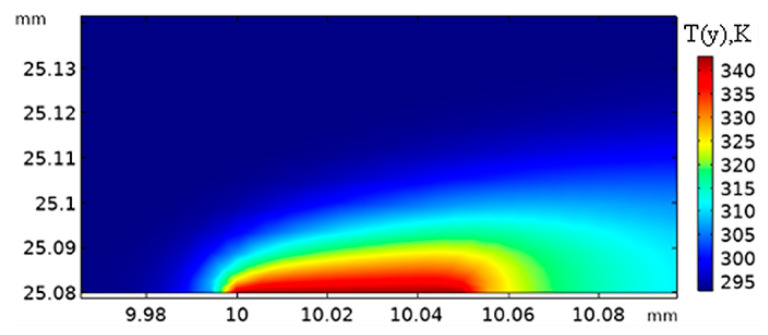
The temperature gradient of a dual-layer hot-film sensor at *τ* = 5 Pa.

**Figure 9:**
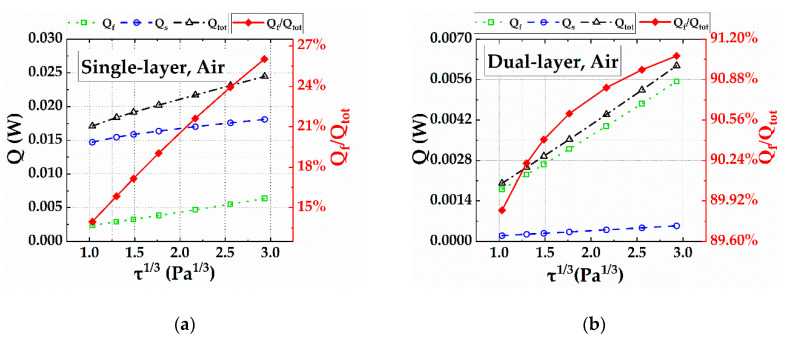
The simulation results of the heat conduction power to the substrate *Q_s_*, the heat transfer power to the fluid *Q_f_*, and the total heat power *Q_tot_* for (**a**) the single-layer hot-film sensor and (**b**) the dual-layer hot-film sensor. The air temperature was 22.5 °C and the two heaters both worked at 45 °C.

The heat transfer processes of a dual-layer hot film sensor were simulated to analyze the heat-insulating effect of the micro dual-layer sensor. The temperature gradient of a dual-layer hot film sensor at *τ* = 5 Pa is shown in Figure 8. The 99% thickness of temperature gradient was 20 μm, which was much less than the thickness of velocity boundary layer (267 μm), indicating that the micro-sensor was consistent with the assumption that the temperature boundary layer should be below the velocity boundary layer. Compared with the single-layer hot film sensor (Figure 9a), the heat conduction power *Q_s_* of the dual-layer hot film sensor (Figure 9b) was dramatically reduced. The heat transfer power *Q_f_* transferring to the air was about 14% to 25% of the total heat power *Q_tot_* in the single-layer hot film sensor—whereas the heat transfer to the air could reach from 89% to 91% through applying the dual-layer structure. In addition, compared to the data in Figure 7 and Figure 9, the total heating powers *Q_tot_* of the two kinds of sensors obtained by simulation were close to those obtained by experiment, revealing the reliability of the simulation results.

## 5. Conclusions

In this paper, a novel dual-layer hot-film sensor for flow detection was proposed. This sensor was fabricated on a flexible PI foil with two-layer nickel heaters. Meanwhile, the intermediate electrical-insulation layer between the two nickel heaters was obtained by imidizing PAA. By effectively insulating the heat conduction from the sensing heater to the substrate, these proposed dual-layer flexible hot-film sensors could achieve from 53.26% to 110.47% improvement in sensitivities compared to the single-layer hot-film sensors. This work presented and characterized a novel approach for heat conduction insulating, and opened new avenues for research on the flexible sensor for flow shear stress detection.

## Figures and Tables

**Table 1:** The basic parameters of four dual-layer hot film sensors.

Sensing Heater	TCR (ppm/°C)	*R*_20_ (Ω)	Correlation Coefficient *R*^2^	Guarding Heater	TCR (ppm/°C)	*R*_20_ (Ω)	Correlation Coefficient *R*^2^
S1	4373.4	15.40	0.9999	G1	3490.9	7.53	0.9999
S2	4370.0	15.10	1.0000	G2	3484.0	7.57	1.0000
S3	4357.7	15.48	1.0000	G3	3476.9	7.80	0.9999
S4	4358.4	15.66	0.9999	G4	3466.4	7.51	0.9999

**Table 2:** The average sensitivities of the single-layer and dual-layer hot film sensors with the flow shear stress varying from 0 to 30 Pa.

Sensor	Average Sensitivity in Single-Layer (mV/Pa)	Average Sensitivity in Dual-Layer (mV/Pa)	Improvement (%)
S1G1	4.68	9.85	110.47
S2G2	5.37	8.23	53.26
S3G3	4.62	7.89	70.78
S4G4	4.42	9.12	106.33
